# Akathisia as an Extrapyramidal Side Effect of Fluoxetine

**DOI:** 10.7759/cureus.15797

**Published:** 2021-06-21

**Authors:** Ijeoma Ajufo, Tajudeen O Basiru

**Affiliations:** 1 Psychiatry, Northridge Behavioral Health PLLC, San Antonio, USA; 2 Developmental Behavioral Pediatrics, Dell Children's Medical Center, Austin, USA

**Keywords:** akathisia, extrapyramidal side effect, prozac, fluoxetine, restlessness

## Abstract

Fluoxetine is a selective serotonin reuptake inhibitor (SSRI) that is commonly prescribed for major depressive disorder (MDD). Akathisia is one of the well-recognized extrapyramidal symptoms (EPS) of antipsychotics and antiemetics, but also a rare manifestation of antidepressants. There are various documentations of EPS of antidepressants including acute dystonia, Parkinsonism, and tardive dyskinesia. Akathisia is not only a rare extrapyramidal manifestation of fluoxetine but a frequently unrecognized phenomenon in those using this medication. This case report describes a case of akathisia observed in a 69-year-old Caucasian female using fluoxetine. Various factors that may have contributed to the development of akathisia in this patient were also discussed as well as implications for clinical practice and future research.

## Introduction

Akathisia is a movement disorder of the extremities characterized by restlessness, inability to lay still, and continuous pacing all of which can be subjective or objective [1, 2]. It is most often an extrapyramidal adverse effect of antipsychotics and antiemetics, but the first case of akathisia was a non-drug-related event observed by a Czech neuropsychiatrist [3, 4]. The definition of akathisia also has a controversial history as some researchers opine that akathisia has a very strong psychological component (subjective) rather than being strictly a movement disorder (objective) with most agreeing that akathisia has both components [5]. Sachdev described the various possible ways that akathisia could present including subjective features like “inner restlessness”, inability to maintain a stable posture like standing, sitting, or lying still, anxiety, irritability, poor concentration or reluctance to take medications as well as worsening of psychotic features [5]. Some objective signs include observable limb movements like leg crossing, lifting, pumping, spontaneously rising from a sitting position, head nodding, shaking, etc. Suicidality has also been observed among many patients with akathisia [6]. Different subtypes of akathisia have been described and include acute, subacute, chronic, pseudo, tardive, and withdrawal akathisia [5].

## Case presentation

Ms. A, a 69-years-old Caucasian female with a history of generalized anxiety disorder and major depressive disorder (MDD) that was being managed with cariprazine by her previous provider presented with extreme restlessness, fidgeting, and pacing around the room for three weeks duration. Two weeks prior to the presentation, her dose of cariprazine was increased from 1.5 mg to 3 mg as the lower dose was not effective in treating depression. She had not responded to a previously prescribed selective serotonin reuptake inhibitor (SSRI), sertraline, which was the reason why she was prescribed cariprazine by her previous psychiatrist. Her depressive symptoms however worsened despite the increased dose of her medication. Around the same time, she experienced other biopsychosocial issues that likely contributed to her depressive symptoms. She was diagnosed with COVID-19, the symptoms of which she thought could be contributing to her depressive symptoms. She also complained that she had been distant from her relatives and none of them had visited her in the preceding months, likely due, in part, to the social distancing requirements of the COVID-19 pandemic. As the pandemic raged, she described herself as a "nervous wreck" and experienced multiple panic attacks. There was no history of mania or hypomania or suicidal thoughts. She was a current cigarette smoker but was not using any other substance.

On an initial mental state examination, she was oriented to time, place, and person, and her speech was fluent. She had congruent affect but her mood was depressed. Blood work did not reveal any significant abnormality. She was diagnosed with akathisia secondary to cariprazine use while the suspected offending drug was discontinued and hydroxyzine was initiated for her panic attacks. The discussion was made to observe her for a few weeks for resolution of her symptoms following discontinuation of cariprazine. Three weeks later, she complained of worsening symptoms of depression although her restlessness, pacing, and panic attacks had improved significantly. We discussed the options available to treat depression given her previous nonresponse to SSRI and reached a conclusion to try another SSRI. Fluoxetine was therefore prescribed and was to be followed up in four weeks. At her follow-up visit, she complained that her akathisia symptoms had returned and were severe enough to keep her awake at night. Fluoxetine was discontinued and a follow-up visit was scheduled. Two weeks later, she reported that her akathisia symptoms had resolved within one week of stopping fluoxetine. Her score on the Naranjo scale was 6. Fluoxetine was documented as the most likely cause of her relapsed akathisia, with the possibility of age and drug interaction being associated factors. Agitated depression was a differential diagnosis but ruled out because the symptoms did not return before the initiation of fluoxetine.

## Discussion

The pathophysiology of akathisia, like other extrapyramidal symptoms (EPS), is thought to be due to dysfunction of the basal ganglia and other striatal pathways although the exact mechanism of the development of akathisia is yet to be agreed upon by the research community [7]. Some of the different postulations for the development of akathisia include dysregulation of the dopaminergic and serotonergic pathways [8], overstimulation of the locus ceruleus [8], blockade of some dopamine receptors in two subdivisions of the ventral striatum [9], and some inflammation involving the blood-brain barriers [10]. There are also possibilities of genetic predispositions according to various genome-wide association studies [11, 12]. Although neuroleptics and anti-dopaminergic antiemetics are primarily indicated in the etiology of drug-induced akathisia, akathisia has also been observed in patients treated with a variety of antidepressant therapy including SSRIs, monoamine oxidase inhibitors (MAOIs), tricyclic antidepressants (TCAs), and even electroconvulsive therapy (ECT), carbamazepine, lithium, and nefazodone [5]. Patients that have previously developed akathisia while being treated with antipsychotics are also thought of as having increased susceptibility to the development of akathisia [13].

Some of the risk factors identified in our patient include age (69 years), female gender, and previous exposure to a neuroleptic agent (cariprazine) [14]. While advanced age may be associated with reduced hepatic clearance, thereby allowing greater bioavailability of the medication and its side effects, the association of gender with increased risk is not fully established. Some authors have reported that such association may be due to the fact that depression is generally more common among females as well as the higher likelihood of a female patient to seek medical treatment than a male patient [15]. A possible risk for the development of akathisia in our patient is a drug interaction. Fluoxetine is known to exacerbate EPS symptoms of antipsychotic agents through various mechanisms such as increasing their plasma concentration and interrupting the serotonin/dopamine balance. Although the patient in this case report was on cariprazine, an antipsychotic agent that presumably caused her initial akathisia, the fact that her akathisia returned and remained severe after discontinuation of cariprazine and initiation of fluoxetine, as well as complete resolution of akathisia symptoms after discontinuation of fluoxetine, made fluoxetine the likely cause of her relapsed akathisia. The patient's score of 6 on the Naranjo scale provided additional support for this conclusion [16]. The fact that the two drugs were not administered concurrently also made drug interaction less likely to be the sole cause of her akathisia. Although cariprazine could have remained in her circulation while fluoxetine was administered, the number of weeks (about seven weeks) that elapsed after discontinuation of cariprazine made this alternate explanation unlikely.

Fluoxetine is an SSRI used in the treatment of MDD, as well as other neuropsychiatric conditions like obsessive-compulsive disorder (OCD), premenstrual dysphoric disorder, panic attacks, bulimia nervosa, and premature ejaculation. According to the National Library of Medicine, fluoxetine is among the most prescribed antidepressants in the United States [17] and antidepressant prescription has gone up in recent years [18, 19]. Fluoxetine potently inhibits 5-hydroxytryptamine (serotonin) reuptake like other SSRIs, although is less selective for serotonin reuptake than other SSRIs. Fluoxetine, in addition to treating depression, is found to improve glucose intolerance in diabetic patients. Like other SSRIs, the most serious adverse effects other than common ones like nausea, vomiting, agitation, headache, malaise, insomnia, and drowsiness include sexual dysfunction, body weight changes, serotonin syndrome, suicide ideation, etc. EPS like akathisia are reportedly rare, occurring in less than one in 1000 patients on fluoxetine [19].

The association of fluoxetine with akathisia and other EPS is not clear-cut. While some authors have assumed that akathisia is commonly induced by fluoxetine [13], other authors that have done extensive work on EPS of antidepressants have cautioned about the assumption of the causal relationship between fluoxetine and akathisia. Leo, for instance, enumerated some factors that “limit the ability to draw firm conclusions about a causal relationship between SSRIs use and the emergence and/or exacerbation of movement disorders”, such as ambiguity of many case reports describing akathisia as a side effect of fluoxetine, a limited number of cases reporting SSRIs as the sole agent being used by patients that report akathisia, existing neurologic disorders in many of these patients as well as the rarity of SSRIs rechallenges [20]. Some of these factors listed by Leo were also seen in our patient, however, the higher likelihood of fluoxetine being the cause of relapsed akathisia in our patient makes this case unique as it will add to the body of evidence describing the rare occurrence of akathisia in patients using antidepressants [21].

## Conclusions

Akathisia is a common EPS of antipsychotics and antiemetics. It is also an uncommon adverse effect of fluoxetine, a commonly prescribed antidepressant, and presents with restlessness, pacing, fidgeting, and sometimes suicidal ideation. Older age, female gender, and previous exposure to antipsychotics are some risk factors that should be looked out for when prescribing Prozac for the treatment of depression. Although akathisia is common among patients being treated with antipsychotics, it is still largely misdiagnosed/underdiagnosed among patients using antidepressants. Our case study is important to increase awareness about antidepressant-induced akathisia.
